# Unpacking the Dynamics of AI-Based Language Learning: Flow, Grit, and Resilience in Chinese EFL Contexts

**DOI:** 10.3390/bs14090838

**Published:** 2024-09-19

**Authors:** Xiuwen Zhai, Ruijie Zhao, Yueying Jiang, Hanwei Wu

**Affiliations:** 1 School of Foreign Language Studies, Guangxi University, Nanning 530004, China; 2 Center for Literature and Culture Studies, Guangxi University, Nanning 530004, China; 3 School of Foreign Studies, Xi’an Jiaotong University, Xi’an 710049, China; ruijiezhao@stu.xjtu.edu.cn; 4 Institute for Advanced Studies in Humanities and Social Science, Beihang University, Beijing 100191, China; jiangyueying@buaa.edu.cn; 5 School of Foreign Studies, Hunan Normal University, Changsha 410000, China

**Keywords:** AI-based language learning, self-determination theory, grit, flow, resilience, Chinese EFL students

## Abstract

Artificial intelligence and positive psychology play crucial roles in education, yet there is limited research on how these psychological factors influence learners’ use of AI, particularly in language education. Grounded in self-determination theory, this study investigates the factors influencing Chinese English learners’ intention to use AI for language learning. Utilizing structural equation modeling, this research examines the mediating roles of grit, flow, and resilience in the relationship between basic psychological needs and the intention to use AI. Data were analyzed using AMOS 26 and SPSS 26. The findings reveal that flow, grit, and resilience mediate the relationship between basic psychological needs and the intention to adopt AI tools for language learning. This study provides valuable insights into how educational environments can be designed to fulfill psychological needs, thereby fostering greater engagement and acceptance of AI in language education.

## 1. Introduction

Postsecondary education, encompassing undergraduate and graduate studies, plays a critical role in equipping individuals with advanced knowledge and skills necessary for personal and professional development [1]. As higher education institutions strive to enhance learning outcomes and adapt to technological advancements, integrating innovative tools becomes essential [2]. Recent advancements in artificial intelligence (AI) have revolutionized several sectors, including education, by providing innovative tools and methods for improving learning processes [3,4]. As educators seek to maximize the benefits of AI, positive psychology plays an important role in its effective use [5,6,7,8]. However, how these positive psychological factors influence learners’ acceptance and sustained use of AI in educational settings remains underexplored. Understanding these factors is crucial for several reasons. Firstly, it can help educators and policymakers design more effective educational programs that leverage AI’s potential. Secondly, this study’s focus on psychological needs, grit, flow, and resilience provides a comprehensive framework for examining how these factors interact to enhance learning engagement and technology adoption. Finally, the findings can inform the development of supportive learning environments that foster essential traits like perseverance, engagement, and adaptability, ultimately leading to more effective and sustainable AI-assisted language learning strategies. This research thus holds significant implications for advancing educational practices and optimizing the integration of AI in language learning.

In this study, self-determination theory was applied in order to examine the factors that affect the intention of Chinese English learners to utilize artificial intelligence for language learning. This study primarily focuses on large language models, such as ChatGPT and Wenxin Yiyan, to explore how these sophisticated AI technologies influence the psychological needs and learning motivations of Chinese English learners. The research employed structural equation modeling to explore how grit, flow, and resilience mediate the relationship between basic psychological needs (BPNs) and the intention to use AI. In this research, three key areas of innovation have been identified: Firstly, it explores the application of artificial intelligence in the field of language education, an area with significant potential yet limited empirical research. Secondly, the study employs a quantitative approach, providing robust statistical evidence to support its findings. Thirdly, it uniquely examines the mediating roles of grit, flow, and resilience, offering a deeper understanding of how these psychological constructs influence learners’ engagement with AI tools. A number of insights gained from these contributions will prove valuable to educators and policy makers wishing to enhance the effectiveness of artificial intelligence-assisted language learning.

## 2. Literature Review

### 2.1. Technology in Language Learning

In the realm of language education, technological advancements have profoundly transformed teaching and learning methodologies [2,9,10,11,12]. The incorporation of technology into the field of language education has significantly revolutionized the methods of teaching and learning languages [2,12]. Over the past two decades, numerous studies [13,14] have explored the impact and effectiveness of various technological tools in enhancing language learning experiences. Previous studies [2,12] have indicated that technology-enhanced language learning (TELL) can significantly improve learning outcomes and offer more efficient methods.

For instance, Yang and Chen [15] found that tools like games and virtual reality (VR) create engaging experiences for learners. Warschauer [16] demonstrated how collaborative tools enhance communication, which is critical for language acquisition. Despite its benefits, integrating technology presents challenges such as technical issues, the need for teacher training, and potential distractions, as highlighted by Yang and Chen [15]. Additionally, Zawacki-Richter et al. [17] explored the potential of cloud computing, natural language processing (NLP), and wearable devices in creating personalized and adaptive learning environments. These technologies can facilitate large-scale collaboration and offer new ways to interact with the target language, making learning more efficient and tailored to individual needs.

Notably, the integration of artificial intelligence (AI) in the field of language education has demonstrated substantial potential [18,19,20,21]. Artificial intelligence-based tools, such as intelligent tutoring systems, chatbots, and virtual reality applications, provide personalized and adaptive instruction. These technologies create interactive learning materials tailored to individual learners’ needs and provide immediate feedback, enhancing the learning experience [15,21,22]. Intelligent tutoring systems adapt to learners’ progress, providing customized support and challenges. These AI technologies have proven effective in improving vocabulary, pronunciation, listening, and speaking skills by providing interactive and engaging learning experiences, increasing motivation, and retention among learners [15,21,22].

Previous research [23,24,25] has utilized artificial intelligence (AI) to investigate the use of technology in language learning. For example, Zhai et al. [25] suggest that AI-driven tools offer strong frameworks for analyzing how learners adopt technology, aiding educators in pinpointing crucial factors that affect engagement in AI-enhanced language learning environments. According to Song and Song [23], AI-assisted language learning, especially when online tools like ChatGPT are used, results in significant improvements in academic writing skills and motivation among English as a Foreign Language students. Their mixed-methods study found that students receiving AI-assisted instruction demonstrated notable improvements in writing proficiency, including organization, coherence, grammar, and vocabulary. Qualitative insights revealed positive perceptions of AI’s role in facilitating immediate feedback and personalized learning experiences, which were instrumental in motivating students to engage more actively in writing tasks. Additionally, Wei [24] posits that AI-assisted language learning significantly boosts learners’ motivation and self-regulated learning. In a study with EFL learners, the experimental group that received AI-mediated instruction was more motivated to learn in the second language and used self-regulated learning strategies more frequently than the control group.

### 2.2. Factors Affecting AI-Assisted Language Learning

#### 2.2.1. Basic Psychological Needs

According to self-determination theory (SDT), the maintenance of social integrity, healthy growth, and successful self-regulation all depend on the satisfaction of the fundamental psychological demands for relatedness, autonomy, and competence [26,27]. These needs are nurtured through supportive environmental and social frameworks [26,27]. Autonomy involves the freedom to make choices and control one’s actions [26,27]. In this research, autonomy is defined as the ability of language learners to use AI to acquire a language while maintaining the freedom to choose vocabulary and settings that suit their individual goals and interests. Competence, defined as the necessity to successfully accomplish tasks or activities [26,27], reflects students’ capacity to acquire and excel in new language skills through engagement with AI, thereby improving their proficiency. Relatedness, the need for connection with others, pertains to learners’ feelings of belonging to a community of language learners, facilitated by shared experiences on the AI-assisted learning platform [28,29]. This theoretical framework, which emphasizes autonomy, competence, and relatedness, offers a comprehensive perspective for understanding the motivational dynamics involved when learners engage with AI for language learning [28,29].

#### 2.2.2. Grit

Duckworth et al. [30] define grit as a characteristic characterized by persistent effort and a strong passion for achieving long-term objectives. It involves maintaining sustained effort and interest over time, even in the face of challenges and setbacks [31]. Grit consists of two main elements: maintaining consistent interest and persevering in effort [30]. Consistency of interest involves maintaining a steady focus on long-term goals without frequently changing direction, while perseverance of effort relates to persistently exerting effort toward these goals despite encountering obstacles [32]. In the context of language learning, persistence and effort are crucial, making grit an important construct. Research has shown that grit is positively related to L2 performance, motivation, emotions, achievement, and other positive psychological variables [33,34,35]. This indicates that grit not only enhances immediate language learning outcomes but also fosters long-term commitment to language study [31,36]. Therefore, understanding and fostering grit in language learners can lead to more effective and sustained language learning practices.

#### 2.2.3. Flow

According to Csikszentmihalyi [37], flow is the psychological state that results from complete immersion in a task and the loss of awareness of time and outside influences. Looking back, Csíkszentmihályi [37] outlined a theoretical construct highly relevant to second language learning success. The ability to balance a task’s demands with a learner’s abilities is fundamental to the flow experience; obstacles are best tackled with the right kind of competence [38,39]. Challenges that can be overcome increase in tandem with skill growth, resulting in an interactive upward spiral or positive feedback loop [40]. Flow creates a state of “harmoniously ordered” consciousness, where thoughts, actions, and emotions are seamlessly coordinated [37,41]. The flow state is inherently gratifying [42] and requires active emotion regulation [43]. When there is an imbalance between challenge and skill, it can lead to feelings of apathy, boredom, worry, and anxiety [44]. The flow channel exists in the “sweet spot” between anxiety and boredom, where rising skills align with escalating challenges, accompanied by well-regulated emotional engagement. Studies in language learning have shown that achieving a flow state can significantly enhance language acquisition and retention. Learners who frequently experience flow are more likely to engage deeply with the material, leading to improved proficiency and greater enjoyment of the learning process [38,45].

#### 2.2.4. Resilience

Originally, the term “resilience” was employed to describe the ability of individuals to adapt and thrive despite facing adversity [46]. In the emerging field of resilience research, the concept has been understood and defined in various ways. Wagnild and Young [47] describe resilience as a personality trait that reduces the negative impact of stress and aids in adaptation. This trait enables individuals to effectively cope with challenges and bounce back from adversity, maintaining psychological stability and continuing to function well despite difficulties. By fostering resilience, individuals can better manage stress and thrive in demanding situations, ultimately leading to improved overall well-being and success in various aspects of life.

Bobek [48] explained resilience as a gradual developmental journey that unfolds over time, encompassing the ability to adapt to varying situations and cultivate competence in challenging circumstances. In the last twenty years, resilience has garnered significant attention in the field of language education [49,50]. In the context of language learning, resilience enables learners to persist despite the inevitable difficulties and setbacks encountered in mastering a new language [51,52]. It helps them to maintain motivation and continue their efforts, even when progress seems slow or obstacles arise [53]. This persistence and adaptability are crucial for achieving long-term success in language acquisition [54].

### 2.3. Theoretical Framework: Self-Determination Theory

Self-determination theory, formulated by Deci and Ryan, provides a comprehensive framework for understanding human motivation and personality [55]. This theory suggests that individuals possess inherent psychological needs crucial for their psychological development, integrity, and well-being [26,56]. These needs encompass autonomy, competence, and relatedness [26,27]. Autonomy is the need to feel in control of one’s actions and objectives. Competence involves the need to master tasks and acquire new skills. Relatedness refers to the desire to connect with others and to both give and receive care and affection. According to SDT theory, fulfilling these fundamental psychological needs enhances intrinsic motivation, leading to greater engagement and persistence in tasks [57]. The theory distinguishes between different types of motivation, emphasizing that intrinsic motivation leads to better learning outcomes, higher engagement, and greater overall well-being compared to extrinsic motivation [58].

SDT posits that when individuals’ fundamental psychological needs are satisfied, they tend to develop self-motivation, which fosters perseverance and passion for long-term goals, commonly referred to as grit. The fulfillment of autonomy, competence, and relatedness enhances an individual’s sense of purpose and determination, which are crucial for grit. This is particularly relevant in AI-assisted language learning, where sustained effort is necessary for mastering new technologies. Therefore, nurturing grit through these needs makes it a logical mediator between psychological needs and the intention to use AI tools. Additionally, flow, or the state of being fully immersed and engaged in an activity, is another critical outcome of need satisfaction per SDT. Learners are more likely to experience a state of flow, which boosts their engagement and motivation to use AI tools, when their needs for autonomy, competence, and relatedness are satisfied. Consequently, creating environments that fulfill these needs promotes optimal learning experiences, making flow a reasonable mediator. Achieving flow in AI-assisted learning significantly boosts learners’ willingness to engage with and persist in using AI technologies. Furthermore, resilience, the ability to bounce back from challenges, is another outcome that SDT explains. Fulfilling basic psychological needs strengthens individuals’ capacity to handle stress and overcome obstacles. In AI-assisted language learning, resilience is crucial for adapting to new technologies and overcoming challenges. Thus, by ensuring learners’ needs for autonomy, competence, and relatedness are met, educators can foster resilience, making it a logical mediator between psychological need fulfillment and the intention to use AI tools, emphasizing the importance of supportive learning environments for sustained technology use.

In summary, self-determination theory provides a robust and comprehensive framework for understanding the motivational processes underlying the use of AI in language learning. The theory’s emphasis on the fulfillment of basic psychological needs aligns well with the mediators identified in the study: grit, flow, and resilience. By meeting learners’ needs for autonomy, competence, and relatedness, educators can cultivate these mediators, thereby enhancing learners’ intention to adopt and persist with AI tools. SDT provides both theoretical foundations and practical insights for the study, highlighting how educational environments can be structured to enhance motivation and engagement in AI-assisted learning.

### 2.4. The Hypothesis Model

**Figure 1:**
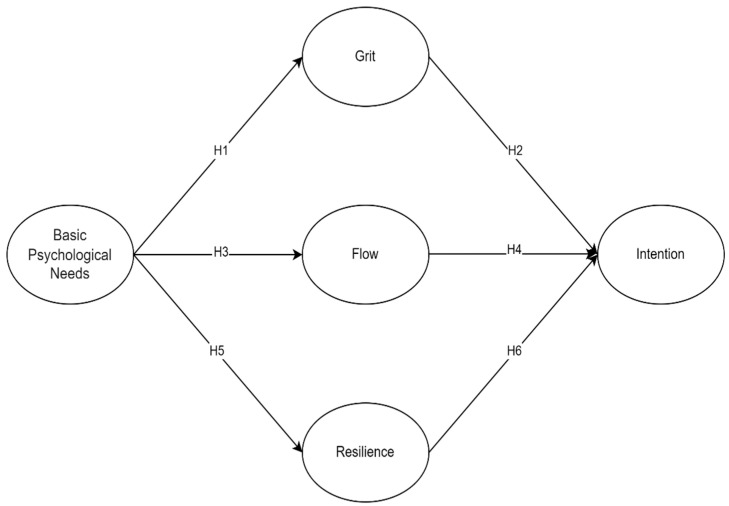
The hypothesis models.

Research has shown that basic psychological needs significantly predict grit. Studies by Huescar Hernandez et al. [59], Shirvan and Alamer [60], and Santana-Monagas and Núñez [61] have demonstrated that when learners perceive their psychological needs for autonomy, competence, and relatedness are met, they develop higher levels of grit, characterized by passion and perseverance. Furthermore, grit has been found to predict the intention to use AI in learning. Gao et al. [62] and Chen Hsieh and Lee [63] highlighted that learners with higher levels of grit are more likely to engage persistently with learning technologies, indicating that grit can influence technology acceptance. Hence, this study also hypothesizes that grit predicts the intention to use AI for language learning. Based on these findings, this study hypothesizes the following (see Figure 1):

**H1.**  *Basic psychological needs predict grit*.

**H2.**  *Grit predicts the intention to use AI for language learning*.

Research indicates that basic psychological needs (BPNs) are significant predictors of flow. Studies by Schüler and Brandstätter [42] and Coterón et al. [64] have shown that fulfilling learners’ needs for autonomy, competence, and relatedness enhances their likelihood of experiencing flow, which is marked by deep immersion and enjoyment in activities. Additionally, flow has been found to influence the intention to use AI in learning. Hu et al. [65] and Tai et al. [66] highlighted that learners who experience flow are more likely to have positive learning outcomes and greater acceptance of technology. Based on these findings, this study hypothesizes the following:

**H3.**  *Basic psychological needs predict flow*.

**H4.**  *Flow predicts the intention to use AI for language learning*.

Research has shown that basic psychological needs (BPNs) significantly predict resilience. Studies by Liu and Huang [39] and Neufeld and Malin [67] have demonstrated that when learners’ psychological needs for autonomy, competence, and relatedness are met, they develop higher levels of resilience, enabling them to effectively cope with challenges. Furthermore, resilience has been found to predict the intention to use AI in learning. Lee and Hancock [68], Versteeg et al. [69], and Zheng et al. [70] highlighted that resilient learners are more likely to adapt positively and engage with new technologies. Based on these findings, this study hypothesizes the following:

**H5.**  *Basic psychological needs predict resilience*.

**H6.**  *Resilience predicts the intention to use AI for language learning*.

## 3. Methodology

### 3.1. Participants

The study included a total of 329 participants, all of whom were recruited through an online questionnaire. These participants were actively pursuing undergraduate or graduate degrees at various higher education institutions, ensuring that the sample accurately represents the postsecondary education population. The inclusion criteria were twofold: first, participants must have used AI for language learning. Specifically, the AI referred to in this study embodies large language models such as ChatGPT and Wenxin Yiyan, which are used to facilitate language learning through interactive and adaptive conversational capabilities. Second, they must have willingly agreed to participate after being fully informed about the study’s purpose, procedures, and intended use of the research findings. In terms of gender, 40.43% are male and 59.57% are female. Regarding age distribution, 23.10% are 19 and below, 56.23% are between 20 and 22, and 20.67% are 23 and above. For university level, 18.24% are from 985 institutions, 24.62% from 211 institutions, and 57.14% are from other institutions. Concerning the field of study, 29.79% are in Arts and Humanities, 28.27% in Social Sciences, 14.59% in Natural Sciences, 18.84% in Engineering, and 8.51% in other fields.

### 3.2. Ethics Statement

This study obtained approval from the Guangxi University Ethics Committee for Research Involving Human Subjects on 6 May 2024 (GXU-2024-048). The Guangxi University Ethics Committee’s stance and decisions are based on the principles of the Declaration of WMA (2021). All subjects involved in this study provided written informed consent.

### 3.3. Instruments

#### 3.3.1. Basic Psychological Needs

In this research, we assessed fundamental psychological needs utilizing a modified version of the questionnaire initially created by Ryan and Deci [71,72] and later modified by Wang and Reynolds [28]. This instrument employs a five-point Likert scale ranging from “strongly disagree” to “strongly agree” to measure participants’ agreement with different statements. It encompasses three primary dimensions: perceived autonomy, perceived competence, and perceived relatedness, with each dimension represented by three items, for a total of nine questions. The perceived autonomy dimension includes items that measure the extent to which participants feel they have control over their actions and decisions. Perceived competence involves items that evaluate participants’ feelings of effectiveness and mastery in their activities. Perceived relatedness assesses the degree to which participants feel connected and valued in their relationships with others. This comprehensive approach ensures a thorough evaluation of the participants’ psychological needs, providing valuable insights into how these needs are being met within the context of the study. By using a well-established and validated instrument, we aim to achieve reliable and accurate measurements that can inform our understanding of the role of psychological needs in various outcomes.

#### 3.3.2. Grit

In this study, we evaluated grit using a modified version of the questionnaire created by Duckworth and Quinn [73], which was customized to align with our research objectives. The instrument utilizes a five-point Likert scale, ranging from “strongly disagree” to “strongly agree”, to evaluate participants’ responses to different statements. Concentrating on a single dimension, the questionnaire comprises eight specific items. These items are designed to capture the persistence and passion for long-term goals that characterize grit. For instance, some questions focus on the participants’ ability to maintain effort and interest over extended periods, while others assess their consistency in working toward their objectives despite challenges and setbacks. By customizing the questionnaire specifically for AI-assisted language learning, we aim to accurately measure how grit influences the learning process in this technologically enhanced environment. These adjustments ensure that the tool is not only relevant but also effective in evaluating the unique aspects of grit that are pertinent to our study. The insights gained from this evaluation will contribute to a better understanding of the role of grit in educational settings, particularly in relation to the integration of AI technologies.

#### 3.3.3. Flow

In this study, we assessed flow utilizing a modified version of the questionnaire created by Smith et al. [74], which was tailored to align with the specific objectives of our research. The instrument employs a five-point Likert scale, ranging from “strongly disagree” to “strongly agree”, to measure participants’ levels of agreement with different statements. Focusing on a single dimension, the questionnaire comprises five specific items designed to measure the state of flow. These items evaluate aspects such as the participants’ immersion in the task, their sense of control over the learning process, the clarity of their goals, and the balance between the challenge of the task and their own skill level. By customizing the questionnaire for AI-assisted language learning, we ensure that it accurately captures the nuances of experiencing flow in this setting. These modifications are essential for obtaining valid and reliable data on how well participants achieve a flow state while engaging with AI tools for language learning. The insights derived from this assessment will provide valuable information on the effectiveness of AI in facilitating immersive and engaging learning experiences, thereby contributing to the broader understanding of flow in educational technology environments.

#### 3.3.4. Resilience

In this study, we assessed resilience utilizing a modified version of the questionnaire created by Kline [75], which was tailored to meet our particular research objectives. The instrument employs a five-point Likert scale, ranging from “strongly disagree” to “strongly agree”, to gauge participants’ responses to various statements. Focusing on a single dimension, the questionnaire includes six specific items designed to measure different aspects of resilience. These items explore participants’ ability to bounce back from challenges, their persistence in the face of adversity, and their capacity to maintain a positive outlook despite difficulties. By tailoring the questionnaire to the context of AI-assisted language learning, we ensure that it accurately captures the nuances of resilience in this particular setting. These modifications are crucial for obtaining valid and reliable data on how students develop and exhibit resilience while engaging with AI tools for language learning. The insights derived from this assessment will provide valuable information on the role of resilience in enhancing learning outcomes and adapting to new educational technologies. Ultimately, this study aims to contribute to a deeper understanding of how resilience interacts with AI-assisted learning environments to support student success.

#### 3.3.5. Intention

In this study, we assessed intention utilizing a modified version of the questionnaire created by Kline [75] and subsequently revised by Wang and Reynolds [28]. The instrument employs a five-point Likert scale, ranging from “strongly disagree” to “strongly agree”, to measure participants’ agreement with various statements. Focusing on a single dimension, the questionnaire comprises three specific items designed to capture the participants’ intention to use AI in their language learning. These items explore aspects such as the participants’ willingness to incorporate AI tools into their study routines, their perceived benefits of using AI for language acquisition, and their likelihood of recommending AI-assisted learning to others. By customizing the questionnaire to align with the specific research objectives, we ensure that it accurately reflects the participants’ attitudes and intentions towards AI in the educational context. These modifications are crucial for obtaining valid and reliable data on the factors that influence the intention to use AI in language learning, providing valuable insights into how technology can be effectively integrated into educational practices. In conclusion, this study aspires to deepen our understanding of the role of intention in the adoption of AI technologies for language learning, highlighting the potential benefits and challenges associated with their use.

### 3.4. Data Analysis

Data analysis for this study was conducted using SPSS 26 and AMOS 26 software, following a detailed four-step process to guarantee a comprehensive examination of the data. First, we meticulously screened for invalid questionnaires, identifying and excluding those with uniformly selected responses or those completed in a time frame too brief to suggest genuine engagement. This step was crucial to maintain the integrity and quality of the data. Second, we conducted descriptive analyses to provide a detailed overview of the distribution and variability of items related to basic psychological needs (BPNs), resilience, flow, grit, and intention. These analyses included measures of central tendency and dispersion, giving us insights into the overall patterns and trends within the data set. Third, we assessed the validity and reliability of the data by calculating Cronbach’s alpha (α) for each variable, which allowed us to determine the internal consistency of our measures. This was followed by a confirmatory factor analysis (CFA) to establish the construct validity of our scales, ensuring that the items accurately represented the underlying theoretical constructs. Finally, a path analysis was performed to investigate the hypothesized relationships among the variables. This advanced statistical technique enabled us to model and test the direct and indirect effects between variables, providing a nuanced understanding of their interactions and the overall structural framework of our study. By following these meticulous steps, we ensured a robust and reliable analysis, leading to valid and actionable findings in the context of AI-assisted language learning.

## 4. Results

### 4.1. Descriptive Analysis

Table 1 presents the descriptive statistics for constructs related to university English learners’ use of AI in language learning, including basic psychological needs, resilience, flow, grit, and intention to use. For basic psychological needs, the mean scores range from 3.55 to 3.69, with standard deviations around 1.10, indicating that learners generally perceive their psychological needs are moderately met while using AI for English learning, with some variability in their experiences. Resilience scores range from 3.67 to 3.73, with standard deviations around 1.10, suggesting that learners feel fairly resilient when engaging with AI for language learning, showing a consistent level of adaptability and persistence. Flow scores have means between 3.65 and 3.74, with standard deviations around 1.06, reflecting that learners often experience a state of flow, feeling absorbed and engaged in their AI-assisted learning activities, with relatively low variability. Grit scores are slightly lower, ranging from 3.50 to 3.58, with similar standard deviations, implying that while learners demonstrate determination and perseverance in using AI for learning, there is a moderate consistency in these traits. Finally, intention to use AI has the highest mean scores, ranging from 3.79 to 3.83, with standard deviations around 1.08, showing a strong and consistent willingness among learners to continue using AI for their English studies.

### 4.2. The Reliability and Validity Checks

This section sequentially presents the results of the reliability analysis, multivariate normality assessment, convergent validity, discriminant validity, and the evaluation of the measurement model. To determine the internal consistency reliability of the nine variables, Cronbach’s α values were calculated. The findings revealed Cronbach’s α values of 0.94 for basic psychological needs, 0.92 for resilience, 0.88 for flow, 0.94 for grit, and 0.84 for intention. Each of these values surpassed the recommended threshold of 0.7, as established by Kline [75], indicating that the scales used in the study exhibit sufficient reliability. This high level of reliability ensures that the measurement instruments are consistent and dependable for assessing the constructs of interest in this research.

Next, the multivariate normality and sampling adequacy of the data were evaluated using the Kaiser–Meyer–Olkin (KMO) measure and Bartlett’s test of sphericity. Bartlett’s test of sphericity yielded a significant result (*p* < 0.001), and the KMO value was 0.94, indicating that the data were appropriate for factor analysis [76]. Furthermore, the univariate skewness and kurtosis values for all scale items were computed to assess normality. As shown in Table 1, the absolute values of skewness and kurtosis for all items were below 2, which confirmed the normality of the dataset [77]. These results demonstrate that the data met the necessary assumptions for proceeding with factor analysis, ensuring the validity of subsequent statistical tests and analyses.

Furthermore, confirmatory factor analysis (CFA) was conducted to examine the establishment of validity. Following the CFA procedures recommended by Collier [76], composite reliability (CR) and average variance extracted (AVE) scores were calculated for each factor (see Table 2) to assess convergent validity. The results indicated that all factors had CR values exceeding the advised value of 0.7 and AVE values above the threshold of 0.5, thus confirming the establishment of convergent validity [77,78]. To evaluate discriminant validity, the square roots of the AVE values and inter-factor correlation coefficients for the nine factors were derived and compared (see Table 2). These square root values were higher than the inter-construct squared correlations, further supporting discriminant validity. The results demonstrated that each construct was distinct from the others, which is crucial for ensuring that the measures used in the study are accurate and reliable. This comprehensive assessment of both convergent and discriminant validity underscores the robustness of the measurement model. By confirming that the constructs measured are both reliable and valid, the study establishes a strong foundation for subsequent analysis, ensuring that the interpretations and conclusions drawn are based on sound and reliable data.

To further examine construct validity, a measurement model was constructed using AMOS 26.0. The model fit was assessed by computing six goodness-of-fit indices: chi-square to degrees of freedom ratio (X^2^/df), comparative fit index (CFI), incremental fit index (IFI), root mean square error of approximation (RMSEA), Tucker–Lewis index (TLI), and standardized root mean squared residual (SRMR). As shown in Table 3, the model fit well with the data, as all indices met the recommended benchmark values [76,77,78,79,80]. Specifically, the X^2^/df ratio was within the acceptable range, indicating an appropriate level of model parsimony. The CFI and IFI values exceeded the 0.90 threshold, reflecting a good fit between the hypothesized model and the observed data. The RMSEA value was below 0.08, indicating a reasonable error of approximation in the population, while the TLI also exceeded the 0.90 benchmark, further supporting the model’s fit. Lastly, the SRMR value was below the 0.08 cutoff, indicating that the model’s residuals were well within acceptable limits.

These results confirm that the measurement model is robust and accurately represents the constructs under study, ensuring that the subsequent structural model analysis is based on a solid foundation. The comprehensive evaluation of model fit indices reinforces the validity and reliability of the constructs, thereby supporting the overall integrity of the research findings.

### 4.3. The Hypothesis Test

Table 4 presents the results of hypotheses testing for the paths between basic psychological needs (BPNs), grit, flow, resilience, and intention to use AI for language learning. The path from BPNs to grit shows a significant positive relationship (β = 0.491, *p* < 0.001, *t* = 8.313, S.E. = 0.059), indicating that meeting psychological needs strongly predicts grit. Similarly, the path from BPNs to flow is significant (β = 0.492, *p* < 0.001, *t* = 8.083, S.E. = 0.054), suggesting that fulfilling psychological needs enhances the flow experience. The relationship between BPNs and resilience is also significant (β = 0.398, *p* < 0.001, *t* = 6.766, S.E. = 0.060), indicating that psychological needs fulfillment fosters resilience. Additionally, resilience significantly predicts the intention to use AI (β = 0.337, *p* < 0.001, *t* = 5.958, S.E. = 0.049). Flow is a significant predictor of intention to use AI (β = 0.405, *p* < 0.001, *t* = 6.679, S.E. = 0.060), and grit also significantly predicts intention (β = 0.340, *p* < 0.001, *t* = 5.882, S.E. = 0.050). All hypothesized paths were accepted based on these results.

**Figure 2:**
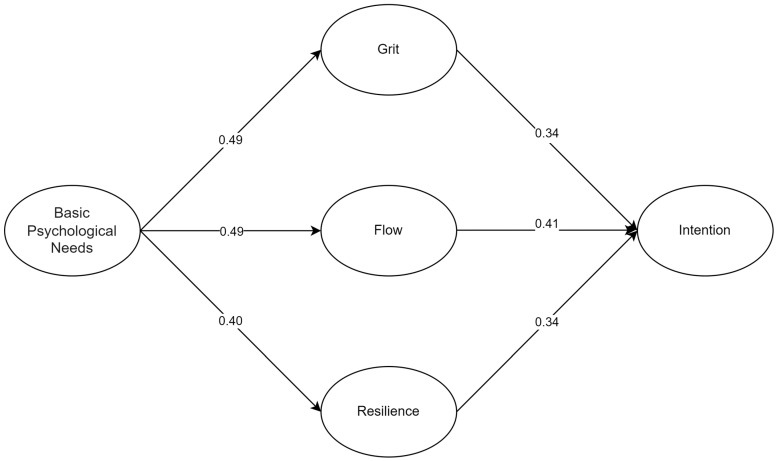
The final model.

Table 5 and Figure 2 present the mediation analysis results for the indirect effects of basic psychological needs (BPNs) on the intention to use AI through grit, flow, and resilience. The indirect effect of BPNs on intention through grit is significant (indirect effect = 0.167, 95% CI [0.107, 0.237], *p* < 0.001), indicating that grit partially mediates this relationship. Similarly, the indirect effect of BPNs on intention through flow is significant (indirect effect = 0.199, 95% CI [0.128, 0.273], *p* < 0.001), suggesting that flow also serves as a mediator in this relationship. Additionally, resilience significantly mediates the relationship between BPNs and intention (indirect effect = 0.134, 95% CI [0.079, 0.196], *p* < 0.001). These results highlight the important roles of grit, flow, and resilience in mediating the effect of basic psychological needs on the intention to use AI for language learning.

## 5. Discussion

This study was designed to investigate the mediating roles of grit, flow, and resilience in the relationship between basic psychological needs and the intention to use AI for language learning. Using structural equation modeling, the study aimed to elucidate how fulfilling learners’ basic psychological needs influences their engagement and willingness to adopt AI tools through these mediators. The discussion section is structured around three key findings, each focusing on the mediation effects of grit, flow, and resilience, respectively. These findings are examined in relation to existing literature, highlighting both theoretical and practical contributions. Specifically, the study found that grit significantly mediates this relationship by emphasizing persistence and passion for long-term goals, which in turn boosts the intention to use AI tools. Flow was shown to mediate by creating intrinsically rewarding experiences that enhance learners’ immersion and engagement. Resilience mediated the relationship by enabling learners to recover from setbacks and maintain consistent effort in using AI for language learning. Additionally, the implications for educational practices and future research directions are discussed, offering valuable insights into enhancing AI-assisted language learning through supportive educational environments. The discussion further elaborates on how educators can foster these mediators by designing learning activities that satisfy basic psychological needs, thereby improving the overall effectiveness of AI-assisted language learning programs.

The mediation analysis revealed that grit significantly mediates the relationship between basic psychological needs and the intention to use AI for language learning. This finding suggests that when learners’ basic psychological needs are met, they develop a stronger sense of perseverance and determination, which in turn enhances their intention to use AI for language learning. This study aligns with the results of Huescar Hernandez et al. [59], Shirvan and Alamer [60], and Santana-Monagas and Núñez [61], all of which emphasize the impact of basic psychological needs on grit. This study extends those findings by applying them to the context of AI tools in language learning. Additionally, our findings are consistent with Gao et al. [62] and Chen, Hsieh, and Lee [63], which highlighted grit as a significant predictor of technology acceptance. The implication here is that educational strategies that aim to satisfy learners’ basic psychological needs can effectively cultivate grit, thereby increasing their intention to adopt and persist with AI tools. This finding contributes to existing research by highlighting grit as a key mediator, emphasizing the importance of fostering grit through psychological need fulfillment to enhance learners’ willingness to adopt AI tools.

The analysis also indicated that flow significantly mediates the relationship between basic psychological needs and the intention to use AI. This suggests that when learners’ basic psychological needs are satisfied, they are more likely to experience a state of flow, characterized by deep absorption and enjoyment in their learning activities, which in turn increases their intention to use AI for language learning. This finding is consistent with the studies of Schüler and Brandstätter [42] and Coterón et al. [64], which emphasized the important influence of basic psychological needs on flow. Moreover, the significant influence of flow on intention is supported by research from Hu et al. [65] and Tai et al. [66], which found that flow experiences enhance learning outcomes and technology acceptance. The significance of this finding lies in its contribution to understanding the role of flow in AI-assisted language learning. It provides empirical evidence that satisfying basic psychological needs can create optimal learning experiences, which are crucial for promoting the sustained use of AI tools. This underscores the potential of educational interventions designed to foster flow states as a means of enhancing technology acceptance and effective learning.

Lastly, the mediation analysis demonstrated that resilience significantly mediates the relationship between basic psychological needs (BPNs) and the intention to use AI for language learning. This indicates that fulfilling learners’ basic psychological needs enhances their resilience, which in turn positively impacts their intention to use AI for language learning. This finding is consistent with Liu and Huang [39] and Neufeld and Malin [67], who emphasized that meeting psychological needs strengthens individuals’ capacity to cope with challenges. Additionally, the significant path from resilience to intention aligns with the findings of Lee and Hancock [68], Versteeg et al. [69], and Zheng et al. [70], which highlighted resilience as a predictor of positive adaptation and engagement with new technologies. This finding extends the understanding of resilience in the context of AI-assisted language learning, underscoring the importance of creating supportive learning environments that fulfill psychological needs to enhance learners’ resilience and their willingness to embrace AI tools. This adds a new dimension to the literature on resilience, highlighting its mediating role in technology acceptance and educational outcomes.

The findings from this study suggest several key practices for educators in postsecondary institutions to enhance AI adoption in language learning. Firstly, educators should design higher education learning environments that support autonomy, competence, and relatedness to cultivate grit among university students [80]. This involves providing undergraduate and graduate students with opportunities to make choices in their learning paths, engage in challenging tasks that align with their advanced skill levels, and foster a sense of community and connection within diverse academic cohorts. Secondly, educators should implement teaching methods and activities tailored to the postsecondary context that promote deep absorption and enjoyment, thereby fostering flow experiences [81,82]. This can be achieved through interactive and immersive AI applications specifically designed for higher education settings, such as gamified learning platforms, virtual reality simulations, and discipline-specific AI tools that make learning both engaging and enjoyable. Lastly, educators in universities and colleges should focus on resilience-building activities and supportive classroom practices to enhance learners’ ability to cope with the unique challenges of postsecondary education. This includes providing constructive feedback on complex assignments, encouraging a growth mindset in research and academic pursuits, and creating a safe and supportive classroom environment that accommodates the diverse backgrounds and experiences of postsecondary students [79,80,83]. By addressing the basic psychological needs of postsecondary learners, educators can strengthen resilience, thereby increasing students’ willingness to embrace and persist with AI tools in their language learning journeys. These practices are essential for creating effective and sustainable AI-assisted learning environments that meet the sophisticated demands of higher education.

This study aligns closely with SDT, which emphasizes the importance of fulfilling basic psychological needs in fostering intrinsic motivation and well-being. Our findings support SDT by demonstrating that when these needs are met, learners develop grit, experience flow, and build resilience, all of which enhance their intention to use AI for language learning. This indicates that creating educational environments that fulfill these basic psychological needs is crucial for promoting sustained engagement with AI tools. The contributions of this study to SDT are twofold. Firstly, it extends the application of SDT to the context of AI-assisted language learning, providing empirical evidence that fulfilling basic psychological needs can enhance learners’ engagement and technology acceptance. Secondly, it identifies grit, flow, and resilience as key mediators in this process, offering a deeper understanding of the mechanisms through which psychological need fulfillment influences learners’ intention to use AI. These insights can inform the design of more effective educational interventions and support the integration of AI tools in language learning environments.

## 6. Conclusions

Grounded in self-determination theory (SDT), this study investigated the factors influencing Chinese English learners’ intention to use AI for language learning. Using structural equation modeling, this research examined the mediating roles of grit, flow, and resilience in the relationship between basic psychological needs and the intention to use AI. The findings revealed that satisfying learners’ basic psychological needs (BPNs) significantly enhances their grit, flow, and resilience, which in turn positively impacts their willingness to adopt AI tools for language learning. Specifically, the study found that when learners feel autonomous, competent, and connected, their persistence (grit), immersive engagement (flow), and ability to recover from challenges (resilience) are strengthened. These psychological mediators then lead to a higher intention to use AI technologies for language acquisition. This study provides valuable insights into how educational environments can be designed to fulfill psychological needs, thereby fostering greater engagement and acceptance of AI in language education. The implications highlight the importance of supporting autonomy, competence, and relatedness to promote sustained use of AI technologies in educational settings.

By creating a learning atmosphere that meets these fundamental needs, educators can enhance students’ intrinsic motivation and commitment to using AI tools, leading to more effective and sustained language learning outcomes. Additionally, this study suggests practical strategies for integrating AI in a way that aligns with SDT principles, such as offering choices in learning activities, providing constructive feedback, and encouraging collaborative learning experiences. These strategies not only improve the immediate learning experience but also contribute to long-term adoption and integration of AI in educational contexts.

This study has several limitations that should be acknowledged. Firstly, being a cross-sectional study, it captures data at a single point in time, which does not account for changes in learners’ attitudes and behaviors over time. This limitation restricts the ability to draw conclusions about causal relationships and the long-term effects of psychological need fulfillment on AI adoption in language learning. Secondly, the study relies solely on quantitative research methods, which may not fully capture the complexity of learners’ experiences and perceptions. Incorporating qualitative data, such as interviews or focus groups, could provide deeper insights into the nuances of how basic psychological needs, grit, flow, and resilience influence the intention to use AI. Furthermore, this study acknowledges that the sample size of 329 participants, while adequate, is not particularly large. This limitation may restrict the generalizability of our findings across different populations or contexts. Future research could address this limitation by employing a larger and more diverse sample size to enhance the representativeness and applicability of the results. Lastly, the exclusive use of quantitative measures may overlook the rich, detailed perspectives that qualitative approaches can reveal. Future research should consider employing longitudinal designs to track changes over time and mixed-method approaches to integrate both quantitative and qualitative data. This combination would address the current study’s limitations and provide a more comprehensive understanding of the factors influencing AI adoption in language learning. As a result, future studies would be able to more effectively capture the dynamic and multifaceted nature of learners’ interactions with AI technologies.

## Figures and Tables

**Table 1:** Descriptive Analysis.

Constructs	Items	M	SD	Skewness	Kurtosis
BPNs	S1	3.67	1.10	−0.30	−0.93
	S2	3.69	1.08	−0.49	−0.49
	S3	3.59	1.09	−0.43	−0.58
	S4	3.59	1.08	−0.39	−0.58
	S5	3.58	1.09	−0.46	−0.40
	S6	3.61	1.09	−0.34	−0.73
	S7	3.62	1.11	−0.44	−0.62
	S8	3.63	1.16	−0.55	−0.53
	S9	3.55	1.17	−0.43	−0.68
Resilience	R1	3.71	1.06	−0.58	−0.28
	R2	3.67	1.09	−0.54	−0.43
	R3	3.68	1.11	−0.49	−0.62
	R4	3.69	1.14	−0.62	−0.42
	R5	3.70	1.15	−0.61	−0.43
	R6	3.73	1.13	−0.60	−0.48
Flow	F1	3.72	1.06	−0.51	−0.47
	F2	3.68	1.04	−0.46	−0.38
	F3	3.74	1.06	−0.66	−0.12
	F4	3.65	1.05	−0.48	−0.40
	F5	3.73	1.06	−0.47	−0.56
Grit	G1	3.54	1.13	−0.40	−0.70
	G2	3.50	1.12	−0.34	−0.73
	G3	3.51	1.12	−0.35	−0.64
	G4	3.51	1.17	−0.39	−0.74
	G5	3.57	1.17	−0.46	−0.73
	G6	3.56	1.13	−0.44	−0.58
	G7	3.55	1.13	−0.36	−0.68
	G8	3.58	1.06	−0.26	−0.79
Intention	I1	3.80	1.08	−0.65	−0.34
	I2	3.79	1.08	−0.62	−0.27
	I3	3.83	1.10	−0.74	−0.18

**Table 2:** The reliability and validity checks.

AVE	CR	α		BPNs	Resilience	Flow	Grit	Intention
0.63	0.94	0.94	BPNs	0.795				
0.66	0.92	0.92	Resilience	0.398	0.812			
0.60	0.88	0.88	Flow	0.492	0.196	0.772		
0.65	0.94	0.94	Grit	0.491	0.195	0.241	0.803	
0.64	0.84	0.84	Intention	0.5	0.483	0.553	0.503	0.798

**Table 3:** Goodness-of-fit indices of the measurement model.

	X^2^/df	CFI	IFI	RMSEA	TLI	SRMR
Our Model	1.795	0.95	0.95	0.03	0.95	0.07
RV	<5	>0.90	>0.90	<0.10	>0.90	<0.08

**Table 4:** Hypothesis test results.

Paths	β	*p*	*t*	S.E.	Results
BPNs-->Grit	0.491	***	8.313	0.059	Accepted
BPNs-->Flow	0.492	***	8.083	0.054	Accepted
BPNs-->Resilience	0.398	***	6.766	0.06	Accepted
Resilience-->Intention	0.337	***	5.958	0.049	Accepted
Flow-->Intention	0.405	***	6.679	0.06	Accepted
Grit-->Intention	0.340	***	5.882	0.05	Accepted

Note: *** = *p* < 0.001.

**Table 5:** The mediation analysis.

Paths	Indirect Effect	95% Confidence Interval		*p*
		Lower	Upper	
BPNs-->Grit-->Intention	0.167	0.107	0.237	***
BPNs-->Flow-->Intention	0.199	0.128	0.273	***
BPNs-->Resilience-->Intention	0.134	0.079	0.196	***

Note: *** = *p* < 0.001.

## Data Availability

The data presented in this study are available upon request from the corresponding author.
